# Myasthénie juvénile oculaire en Afrique Subsaharienne: cas de deux sœurs germaines issues d’un mariage consanguin au Togo

**DOI:** 10.11604/pamj.2017.28.63.13709

**Published:** 2017-09-21

**Authors:** Nidain Maneh, Kossivi Apetse, Bénédicte Marèbe Diatewa, Sidik Abou-Bakr Domingo, Aidé Isabelle Agba, Koffi Didier Ayena, Koffi Agnon Balogou, Komi Patrice Balo

**Affiliations:** 1 Université de Lomé, Faculté des Sciences de la Santé, Lomé, Togo; 2 Service d’Ophtalmologie, CHU-Campus de Lomé, Lomé, Togo; 3 Service de Neurologie, Chu-campus de Lomé, Lomé, Togo

**Keywords:** Sub-Saharan Africa, consanguinity, juvenile myasthenia gravis, ptosis, Sub-Saharan Africa, consanguinity, juvenile myasthenia gravis, ptosis

## Abstract

La myasthénie est une pathologie auto-immune acquise rare à l'origine de la déficience de la transmission neuro-musculaire dont la forme juvénile se manifeste souvent par une atteinte oculaire exclusive. Nous rapportons deux cas de myasthénie juvénile oculaire (MJO) au sein d'une même fratrie. il s'agit de deux fillettes XA et XB respectivement âgées de 11 et 9 ans d'origine malienne résidant au Togo, issues d'un mariage consanguin de 1^er^ degré, amenées en consultation d'ophtalmologie, pour une baisse progressive de l'acuité visuelle (AV). XA avait aux deux yeux, une AV à 8/10 et XB, une AV de 3/10 améliorée au trou sténopéïque à 7/10 en faveur d'une amétropie. XA présentait un ptosis bilatéral qui remontait à l'âge de 2 ans avec une action du releveur de la paupière supérieure (RPS) à 7 mm et XB, un ptosis bilatéral remontant à l'âge de 3 ans avec une action du RPS nulle. Pour les deux, le test au glaçon était franchement positif, le signe de Cogan présent avec une parésie oculo motrice sans diplopie. Le dosage des auto-anticorps anti récepteurs de l'acéthylcholine était normal. Le diagnostic de MJO associée à une amétropie a été évoqué. Une correction optique de l'amétropie par des lunettes et un traitement spécifique par la pyridostigmine avait été instaurée mais les patientes ont été perdues de vue. la myasthénie auto-immune avec des manifestations ophtalmologiques inaugurales est rare mais possible chez l'enfant en Afrique subsaharienne où des études sont nécessaires pour y déterminer les éventuelles particularités de la maladie.

## Abstract (en)

Myasthenia gravis is a rare acquired autoimmune pathology causing neuromuscular transmission impairment. Juvenile onset of myasthenia gravis is often characterized by ocular involvement. We report two cases of ocular juvenile myasthenia gravis (JMG) in two siblings. They were two young girls, XA and XB, aged 11 and 9 years, of Malian origin, residing in Togo, born from first-degree of consanguinity presenting to Ophthalmology due to progressive decrease in visual acuity. XA showed visual acuity 8/10 on both eyes while XB showed improvement in visual acuity from 3/10 to 7/10 using a pinhole occluder, suggesting ametropia. XA had a 2-year history of bilateral ptosis lifting the upper eyelid of 7 mm, while XB had a 3-year history of bilateral ptosis with no lifting of the upper eyelid. Ice pack test was strongly positive in both patients. They had Cogan's lid twitch with paresis of the oculomotor nerve without diplopia. The dosage of acetylcholine receptor autoantibodies was normal. The diagnosis of JMG associated with ametropia was suspected. Ametropia was corrected by glasses and a specific treatment with pyridostigmine was initiated, but both patients were lost to follow-up. Autoimmune myasthenia gravis with inaugural ophthalmologic manifestation is rare but it can occur among children living in sub-Saharan Africa. Studies should be conducted to establish the features of this disease.

## Introduction

La myasthénie est une pathologie auto-immune acquise rare avec une prévalence de 20 cas par 100000 habitants, à l'origine de la déficience de la transmission neuro-musculaire [1]. Elle est liée à un blocage des récepteurs de la plaque motrice par des anticorps anti récepteurs à l'acétylcholine (Ac anti RAch) [2] ou plus rarement à des anticorps anti tyrosine kinase spécifique du muscle (Ac anti MuSK). Le diagnostic de myasthénie repose sur un faisceau d'arguments cliniques recueillis à l'interrogatoire et à l'examen clinique ainsi que sur des examens paracliniques (électroneuromyogramme, ENMG et dosage des anticorps spécifiques) [3]. Chez l'enfant, on distingue la myasthénie congénitale en lien avec des anomalies génétiques apparaissant dès la naissance, la myasthénie néonatale qui est transitoire liée à un transfert transplacentaire des anticorps maternels anti RAch et enfin, la myasthénie juvénile (MJ) caractérisée par un début à l'âge prépubertaire [4]. La MJ se manifeste le plus souvent par une atteinte oculaire exclusive et est fréquente en Asie [5], rare chez les caucasiens [1] et de fréquence inconnue en Afrique subsaharienne. Des cas de myasthénie familiale ont été rapportés depuis les années 1970 mais rarement en Afrique subsaharienne [6]. Nous présentons deux cas de MJ oculaire chez deux sœurs germaines d'origine malienne résidant au Togo issues d'un mariage consanguin de 1^er^ degré.

## Patient et observation

Il s'agit de deux fillettes de 9 et 11 ans issues d'une fratrie de six enfants, amenées par leur maman en consultation d'ophtalmologie au CHU-Campus de Lomé, pour une baisse progressive de l'acuité visuelle (AV). Elles sont issues d'un mariage consanguin de 1er degré et ne présentent aucun antécédent personnel ou familial particulier.

**Figure 1:**
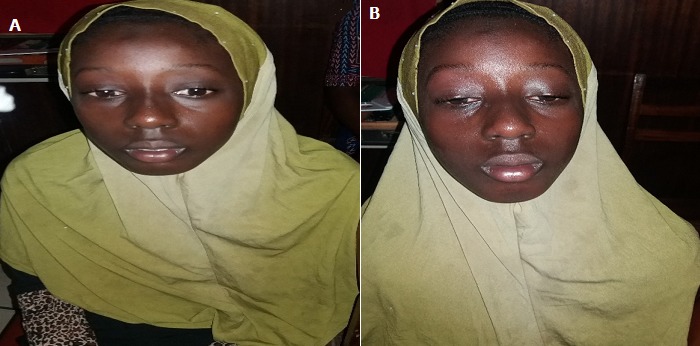
A) XA: ptosis bilatéral; B) XA: ptosis majoré après le regard prolongé en haut (fatigabilité à l’effort)

**Observation 1:** XA, fillette de 11 ans, élève, présente depuis l'âge de deux ans un ptosis bilatéral plus marqué en fin de journée. Une baisse de l'AV est apparue progressivement depuis environ 2 mois. L'interrogatoire ne retrouvait pas de fatigabilité des membres à l'effort ni de troubles d'atteinte bulbaire. L'AV aux deux yeux était à 8/10. On notait un ptosis bilatéral léger, avec une action du releveur de la paupière supérieure (RPS) à 7 mm. Une atteinte des muscles oculomoteurs avec une paralysie complète de l'élévation, une limitation dans l'abduction, l'adduction et dans l'abaissement des globes oculaires était objectivée sans diplopie. Les pupilles étaient de taille et de forme normales. Le test au glaçon était franchement positif avec une disparition du ptosis. Le signe de Cogan était également présent. Une augmentation de l'importance du ptosis dans le regard soutenu en haut avait été observée révélant la fatigabilité à l'effort (Figure 1).

**Figure 2:**
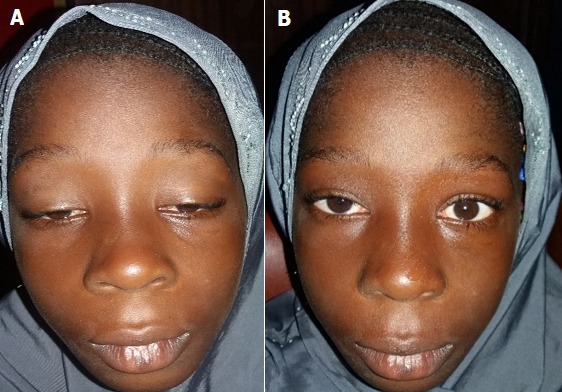
XB: régression du ptosis après le test au glaçon B) XB: ptosis avant le test au glaçon

**Observation 2:** XB, fillette de 9 ans, élève, présente un ptosis bilatéral plus marqué en fin de journée depuis l'âge de trois ans. Un flou visuel d'apparition progressive depuis 3 mois a motivé la consultation. A l'examen ophtalmologique, l'AV aux deux yeux était de 3/10 améliorée au trou sténopéïque à 7/10. On notait un ptosis bilatéral modéré, avec une action du RPS nulle. Une atteinte des muscles oculomoteurs avec une limitation de l'élévation, de l'abduction, de l'adduction et de l'abaissement était présente sans diplopie. Les pupilles étaient de taille et de forme normales. Le test au glaçon était positif en une minute (Figure 2). Le signe de Cogan était présent. Devant ces deux tableaux cliniques nous avons évoqué une myasthénie juvénile oculaire associée à une amétropie. Une évaluation neurologique et générale avait confirmé la localisation exclusivement oculaire du syndrome myasthénique et l'absence de signes généraux d'affection systémique.

**Examens paracliniques:** Les explorations complémentaires ont été limitées car étaient entièrement à la charge des patientes. Le dosage des auto- anticorps anti RAch était normal : 0,1nmol/l pour XA et 0,15nmol/l pour XB. Le bilan thyroïdien, le dosage des Créatine Phospho Kinases et la tomodensitométrie thoracique à la recherche d'un thymome étaient normaux. Une correction optique de l'amétropie par les lunettes et un traitement spécifique par la pyridostigmine avaient été instaurés mais les patientes ont été perdues de vue. Une enquête familiale poussée à la recherche d'autres cas dans la famille, n'a pu être réalisée de ce fait.

## Discussion

Nous avons rapporté deux cas de MJ oculaire dont le diagnostic est basé sur les caractéristiques cliniques. Le ptosis bilatéral fluctuant est un signe évocateur de la myasthénie. Selon Sommer et al. [7], le ptosis fluctuant et asymétrique s'il est bilatéral, avec ou sans diplopie, est révélateur de la myasthénie dans 40 à 50 % des cas. Chez l'enfant, la myasthénie reste le plus souvent oculaire mais peut aussi se généraliser. Dans nos deux cas, la localisation exclusivement oculaire pendant plusieurs années fait évoquer une forme oculaire pure de la myasthénie. La positivité du test au glaçon est un argument majeur pour le diagnostic de myasthénie car sa sensibilité est autour de 95% pour les MO avec une spécificité évaluée à 97% [7]. Ceci compense le défaut de sensibilité des examens complémentaires (Ac anti RACh absents dans 45% des cas, plus de 50% de faux négatifs à l'ENMG) dans cette forme de myasthénie. L'âge de début des symptômes de nos cas est de 2-3 ans et se situe dans la tranche de de 18 mois à 11 ans (avant 3 ans dans 8/18 cas) rapportée par VanderPluy et al. pour les MJ [8]. Le délai de consultation est de plusieurs années chez nos patientes, les parents ayant estimé que le ptosis était constitutionnel pas forcément pathologique. La consultation du spécialiste n'a finalement été faite que devant l'apparition de la baisse d'AV en lien avec une amétropie. De plus, les patientes ont été perdues de vue malgré les multiples relances témoignant d'une non adhésion aux thérapeutiques proposées. Cette méconnaissance de la pathologie myasthénique ne se limite pas à la population mais touche aussi le personnel soignant du fait de la rareté de la maladie. Ainsi, Alam et al. [9] ont rapporté qu'une MO avait été confondue à un ptosis congénital. Du fait de sa curabilité [3] avec des moyens généralement peu couteux, la myasthénie doit faire l'objet d'une sensibilisation permanente pour limiter les risques de sous diagnostic.

La survenue de la myasthénie chez des sœurs germaines issues d'un mariage consanguin plaide pour une implication génétique dans la survenue de ces cas. Les cas de myasthénie au sein d'une même famille ont été rapportés depuis plusieurs années [5]. Des gènes dont le degré de pénétrance est variable ont été identifiés. La consanguinité augmente ainsi le risque de survenue de la maladie chez les descendants. En l'absence d'enquête familiale poussée, il est difficile d'appréhender l'impact des facteurs génétiques dans la survenue de ces cas. A notre connaissance, il s'agit des premiers cas de MJO rapportés au sein d'une même famille en Afrique subsaharienne. Des études complémentaires sont nécessaires pour identifier l'importance et éventuellement la particularité des facteurs génétiques dans la survenue de la myasthénie en Afrique subsaharienne.

## Conclusion

La myasthénie auto-immune est une affection rare mais possible chez l'enfant avec le plus souvent des manifestations oculaires inaugurales orientant les parents vers l'ophtalmologiste. C'est le premier diagnostic à évoquer devant un ptosis bilatéral ou non, fluctuant ou non. Un diagnostic précoce et une prise en charge neurologique permettrait de limiter les risques de généralisation de l'affection pouvant compromettre le pronostic vital. Nos deux cas rapportés confortent l'hypothèse d'une implication génétique dans la survenue de la maladie. Des études sont nécessaires pour déterminer les éventuelles particularités de la maladie en Afrique subsaharienne.

## Conflits d’intérêts

Les auteurs ne déclarent aucun conflit d'intérêts.
